# 2D/2D Phosphorus-Doped g-C_3_N_4_/Bi_2_WO_6_ Direct Z-Scheme Heterojunction Photocatalytic System for Tetracycline Hydrochloride (TC-HCl) Degradation

**DOI:** 10.3390/ijerph192214935

**Published:** 2022-11-13

**Authors:** Xudong Yin, Xiaojie Sun, Dehao Li, Wenyu Xie, Yufeng Mao, Zhenghui Liu, Zhisen Liu

**Affiliations:** 1 Guangxi Key Laboratory of Environmental Pollution Control Theory and Technology, College of Environmental Science and Engineering, Guilin University of Technology, Guilin 541006, China; 2 Guangdong Provincial Key Laboratory of Petrochemical Pollution Processes and Control, Key Laboratory of Petrochemical Pollution Control of Guangdong Higher Education Institutes, School of Environmental Science and Engineering, Guangdong University of Petrochemical Technology, Maoming 525000, China

**Keywords:** P-doped g-C_3_N_4_, Bi_2_WO_6_, 2D/2D direct Z-scheme heterojunction, photocatalytic degradation, tetracycline hydrochloride

## Abstract

Bi_2_WO_6_-based heterojunction photocatalyst for antibiotic degradation has been a research hotspot, but its photocatalytic performance needs to be further improved. Therefore, 2D/2D P-doped g-C_3_N_4_/Bi_2_WO_6_ direct Z-scheme heterojunction photocatalysts with different composition ratios were prepared through three strategies of phosphorus (P) element doping, morphology regulation, and heterojunction, and the efficiency of its degradation of tetracycline hydrochloride (TC-HCl) under visible light was studied. Their structural, optical, and electronic properties were evaluated, and their photocatalytic efficiency for TC-HCl degradation was explored with a detailed assessment of the active species, degradation pathways, and effects of humic acid, different anions and cations, and water sources. The 30% P-doped g-C_3_N_4_/Bi_2_WO_6_ had the best photocatalytic performance for TC-HCl degradation. Its photocatalytic rate was 4.5-, 2.2-, and 1.9-times greater than that of g-C_3_N_4_, P-doped g-C_3_N_4_, and Bi_2_WO_6_, respectively. The improved photocatalytic efficiency was attributed to the synergistic effect of P doping and 2D/2D direct Z-scheme heterojunction construction. The stability and reusability of the 30% P-doped C_3_N_4_/Bi_2_WO_6_ were confirmed by cyclic degradation experiments. Radical scavenging experiments and electron spin resonance spectroscopy showed that the main active species were •O_2_^−^ and h^+^. This work provides a new strategy for the preparation of direct Z-scheme heterojunction catalysts with high catalytic performance.

## 1. Introduction

Tetracycline hydrochloride (TC-HCl) is one of the commonly used antibiotics. It is widely used in human medicine, animal husbandry, and agriculture to prevent disease and promote growth [1,2]. TC-HCl is difficult to be absorbed and utilized by organisms. About 70–90% of unutilized or incompletely utilized TC-HCl is discharged into natural waterbodies and soils. In recent years, it has been frequently detected in surface water, groundwater, soil, vegetables, fruits, and other foods around the world [3,4]. Owing to its good water solubility, easy accumulation and migration, stable structure, poor degradability, bio-resistance, and chronic toxicity, this poses serious harm to natural ecosystems and human health [5]. Therefore, there is an urgent need to develop a safe and effective method of disposing of TC-HCl. Photocatalytic degradation has a high degradation rate and high mineralization rate, environmental friendliness, and the ability to degrade TC-HCl into low-toxic byproducts, namely, CO_2_ and H_2_O; therefore, it has emerged as a promising research topic in recent years [6,7,8].

Bi_2_WO_6_ is a visible light-responsive photocatalyst that is widely used in fields such as energy generation and pollutant degradation owing to its stable physical and chemical properties, narrow band gap, non-toxicity, and low cost [9,10]. However, it has a limited range of visible light absorption, and the photogenerated carriers easily recombine between the W 5d orbital and the hybrid orbitals of Bi 6s and O 2p, which reduces the quantum efficiency of its protons and limits its practical applications [11]. The photocatalytic performance of Bi_2_WO_6_ can be effectively improved by morphological control to produce diverse morphologies, including three-dimensional (3D) nanoflowers [12], two-dimensional (2D) nanosheets [13], one-dimensional (1D) nanofibers [14], and zero-dimensional (0D) quantum dots [15]. Among these, 1D and 0D Bi_2_WO_6_ materials have unsatisfactory photocatalytic performance; and 3D Bi_2_WO_6_ has a large specific surface area with many channels, which is conducive to the adsorption of reactants, but no current synthesis method allows the precise adjustment of its porosity and active sites, and the photocatalytic performance needs to be further improved. In contrast, 2D Bi_2_WO_6_ has shorter charge transport pathways, a larger specific surface area, higher adsorption, and more abundant active sites, which facilitate the separation of photogenerated electron–hole pairs, making it the preferred morphology for morphological control [16]. However, morphological control alone does not solve the issue of easy recombination of photogenerated electrons and holes, which means it is difficult to achieve satisfactory photocatalytic performance.

Combining 2D Bi_2_WO_6_ and other catalysts to form heterojunctions can effectively solve the issue of electron–hole recombination and greatly improve the catalytic performance. Compared with 0D/2D and 1D/2D heterojunction photocatalysts, 2D/2D heterojunctions have higher specific surface areas, lower transport resistance, higher charge transfer efficiency, more active sites, and better photocatalytic performance, making them an important research topic [17]. Depending on the photocatalytic mechanism, 2D/2D Bi_2_WO_6_ heterojunction photocatalysts can be classified into two types, namely, Z-scheme [18] and type-II [19]. Among them, the photogenerated electrons and holes of Z-scheme heterojunctions are more negative and positive, respectively, than these of type-II heterojunctions, and the resulting redox ability is far superior. Therefore, these materials have considerable potential for use in pollutant degradation applications.

The most common types of 2D/2D Bi_2_WO_6_ Z-scheme heterojunctions are all-solid-state materials with a solid medium, such as 2D/2D AgBr/GO/Bi_2_WO_6_ [20] and 2D/2D TiO_2_/Au/Bi_2_WO_6_ [21], and direct Z-scheme heterojunctions without any medium, such as 2D/2D Bi_2_WO_6_/ZnIn_2_S_4_ [22] and 2D/2D WS_2_/Bi_2_WO_6_ [18]. Direct Z-scheme heterojunctions have lower cost, faster photogenerated electron transfer, prevention of reverse reactions, and no light-shielding effect, and they have been proven to be the most promising third-generation heterojunction catalysts [23]. However, there is a need to identify superior co-catalysts to combine with 2D Bi_2_WO_6_ to form 2D/2D direct Z-scheme heterojunctions to guarantee optimal photocatalytic performance.

Graphitic carbon nitride (g-C_3_N_4_), as a non-toxic, inexpensive, and metal-free 2D semiconductor with a graphite-like structure, has been widely used for the photocatalytic degradation of pollutants. However, its rapid photogenerated carrier recombination rate and low visible-light utilization lead to low photocatalytic performance. Methods such as doping and heterojunction formation are commonly used to improve its photocatalytic activity. Since the valence and conduction bands of g-C_3_N_4_ and Bi_2_WO_6_ are well matched, the forming of direct Z-scheme heterojunctions with Bi_2_WO_6_ can greatly improve the photocatalytic performance of g-C_3_N_4_ [24,25,26]. However, the catalytic performance of g-C_3_N_4_/Bi_2_WO_6_ needs to be further improved. In addition, non-metallic elements are often used to modify g-C_3_N_4_ because of their low cost and environmental friendliness. P doping is promising because P atoms can form chemical bonds with adjacent C and N atoms, resulting in planar coordination, enhanced active centers, improved electrical conductivity and charge transfer capabilities, a significant reduction in the band-gap width of g-C_3_N_4_, a broadened range of visible light absorption, and improved photocatalytic performance [27,28,29]. Based on these factors, we hypothesized that 2D/2D P-doped g-C_3_N_4_/Bi_2_WO_6_ heterojunction catalysts, which combine P doping, morphological control, and heterojunction construction, will present excellent photocatalytic performance. At present, there are no reports on the photocatalytic degradation of TC-HCl by P-doped g-C_3_N_4_/Bi_2_WO_6_.

In this study, 2D P-doped g-C_3_N_4_ was prepared by calcination, and samples of 2D/2D P-doped g-C_3_N_4_/Bi_2_WO_6_ were prepared at different ratios using a hydrothermal method. The catalyst was characterized using various techniques, and the catalytic activity toward TC-HCl degradation was evaluated under visible light illumination. Further, the effects of coexisting ions and different water sources on the degradation efficiency were studied, and finally, the reaction pathways and mechanism of photocatalytic degradation were proposed.

## 2. Materials and Methods

### 2.1. Materials

Dicyandiamide and 96% hydroxyethylidene diphosphonic acid (HEDP) were purchased from Shanghai Macklin Biochemical Co., Ltd. (Shanghai, China); bismuth nitrate pentahydrate, sodium bicarbonate, sodium chloride, and sodium sulfate were purchased from Tianjin Kemiou Chemical Reagent Co., Ltd. (Tianjin, China); 96% TC-HCl was purchased from Shanghai Aladdin Biochemical Technology Co., Ltd. (Shanghai, China); 95% ethanol, ascorbic acid, isopropanol, sodium nitrate, and magnesium sulfate were purchased from Xilong Scientific Co., Ltd. (Shantou, China); sodium tungstate dihydrate, ethylenediaminetetraacetic acid disodium salt (EDTA-2Na), calcium sulfate, aluminum sulfate, and manganese sulfate were purchased from Tianjin Damao Chemical Reagent Factory (Tianjin, China); and humic acid was purchased from Tianjin Guangfu Fine Chemical Research Institute (Tianjin, China). All of the above reagents were of analytical grade.

### 2.2. Preparation of P-Doped g-C_3_N_4_

P-doped g-C_3_N_4_ was prepared by a two-step calcination process. Dicyandiamide (15 g) and HEDP (0.5 g) were dissolved in ethanol (30 mL) and then stirred in a water bath at 70 °C until the ethanol evaporated completely. The dried product was placed in a 100 mL covered ceramic crucible and calcined at 550 °C for 4 h in a muffle furnace (heating rate: 3 °C/min). After cooling, the product was ground for 10 min, then placed in a 100 mL uncapped ceramic crucible and calcined at 550 °C for 2 h (heating rate: 3 °C/min). P-doped g-C_3_N_4_ (PCNS) was obtained after cooling. For comparison, g-C_3_N_4_ nanosheets (CNS) were prepared under the same conditions without the addition of HEDP.

### 2.3. Preparation of P-Doped g-C_3_N_4_/Bi_2_WO_6_

P-doped g-C_3_N_4_/Bi_2_WO_6_ was prepared by a hydrothermal method. Na_2_WO_4_·2H_2_O (0.3299 g) and Bi(NO_3_)_3_·5H_2_O (0.9701 g) were dissolved separately in deionized water (30 mL). The two solutions were then mixed (Na_2_WO_4_·2H_2_O/Bi(NO_3_)_3_·5H_2_O molar ratio of 1:2), and PCNS (0.2093 g) was added. Complete mixing was ensured by magnetic stirring three times for 30 min, each with sonication for 30 min between each stirring step. The obtained solution was transferred to a 100 mL Teflon-lined autoclave and held at 170 °C for 20 h. After cooling, the solution was centrifuged at 7500 cycles/min for 10 min, followed by washing with ethanol and deionized water alternately three times. Finally, the sample was dried at 80 °C overnight to obtain 30 wt% P-doped g-C_3_N_4_/Bi_2_WO_6_ (denoted as 30% PCNS/BWO). Samples with 0.5 wt%, 10 wt%, 50 wt%, and 67 wt% PCNS (denoted as 0.5% PCNS/BWO, 10% PCNS/BWO, 50% PCNS/BWO, and 67% PCNS/BWO, respectively) were prepared in the same way using different amounts of PCNS. For comparison, BWO was prepared without the addition of PCNS, and 30% CNS/BWO was prepared by replacing PCNS with CNS. The quantities of the reagents used in the preparation of all catalysts are listed in Table S1.

### 2.4. Characterization of the Catalysts

For each catalyst, the crystal structure was determined using X-ray diffractometry (XRD; Bruker D8); the morphology was determined by scanning electron microscopy (SEM; JEOL JSM-7500F) and transmission electron microscopy (TEM; JEOL JEM-2100F); the surface functional groups and chemical bonds were determined by Fourier-transform infrared spectroscopy (FTIR; Nicolet 460); the optical properties were determined by ultraviolet-visible diffuse reflectance spectroscopy (UV-vis DRS; Shimadzu UV2700); photoluminescence (PL) spectra and fluorescence lifetimes were determined using a spectrofluorophotometer (Edinburgh Instruments FLS980); the elemental composition and valence states were analyzed by X-ray photoelectron spectroscopy (XPS; Thermo Fisher Escalab 250Xi); the specific surface area and pore size distribution were evaluated using the Brunauer–Emmett–Teller (BET) method (Micromeritics ASAP2020); electrochemical impedance spectroscopy (EIS) and photocurrent response curves were obtained using an electrochemical workstation (Shanghai Chenhua, CHI1030B).

### 2.5. Evaluation of Photocatalytic Performance

For each catalyst, 0.02 g of catalyst was added to 100 mL of a 20 mg/L TC-HCl solution, followed by stirring in the dark for 20 min. Thereafter, photodegradation experiments were conducted under the irradiation of a xenon lamp (PLS-SXE300D, Perfect Light, Beijing, China) at 300 W and *λ* > 420 nm. The samples were collected every 15 min and filtered with a 0.22 µm filter. The absorbance of each sample at 357 nm was measured to calculate the degradation efficiency. 

To evaluate the reusability of the 30% PCNS/BWO photocatalyst, the TC-HCl degradation experiment was repeated four times. After each degradation experiment, the catalyst was collected by centrifugation, washed three times with deionized water and ethanol alternately, and dried at 80 °C for the next degradation experiment.

To explore the effects of coexisting ions and different water sources, which influences the practical use of the 30% PCNS/BWO photocatalyst, the effects of humic acid (10 mg/L), 5 mM of different anions and cations (HCO_3_^−^, Cl^−^, NO_3_^−^, SO_4_^2−^, Ca^2+^, Mg^2+^, Al^3+^, and Mn^2+^), and different water sources (river water, final effluent of a sewage treatment plant, lake water, and deionized water) on the TC-HCl degradation efficiency were studied. The experimental conditions were the same as those for the photodegradation experiments.

The intermediates of the TC-HCl photocatalytic degradation process using the 30% PCNS/BWO photocatalyst were identified by high-resolution accurate mass liquid chromatography with tandem mass spectrometry (HRAM LC-MS/MS; Thermo Scientific, Q Exactive). The secondary mass spectra of the intermediates were obtained by comparison with the standard library, and then the mass-to-charge ratios of the intermediates were determined by comparison with similar literature.

### 2.6. Detection of Active Substances

To explore the main active species during the degradation of TC-HCl using the 30% PCNS/BWO photocatalyst, 1 mM ascorbic acid, 1 mM EDTA-2Na, and 1 mM isopropanol (IPA) were added during the photocatalytic process to scavenge superoxide radicals (•O_2_^−^), holes (h^+^), and hydroxyl radicals (•OH), respectively. In addition, the free radicals produced during the degradation process were determined by electron spin resonance (ESR) spectroscopy (Bruker E-500). The experimental conditions were the same as those for the photodegradation experiments.

## 3. Results and Discussion

### 3.1. Characterization

**Figure 1:**
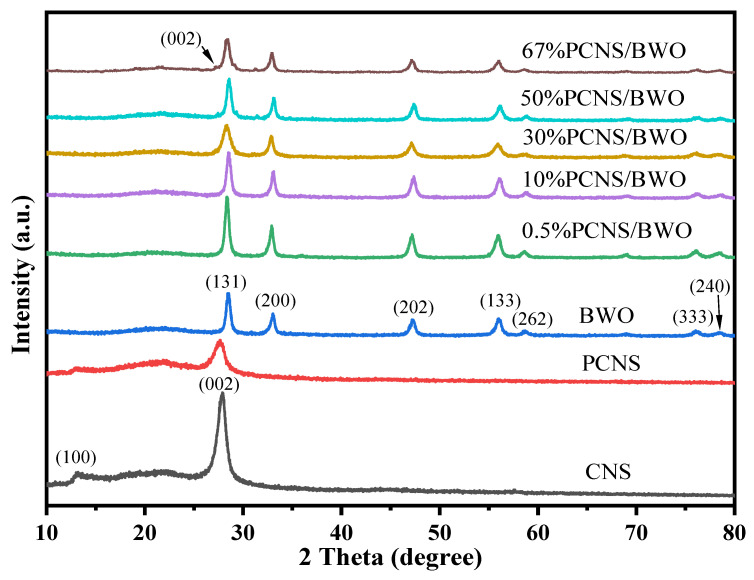
XRD patterns of the synthesized samples.

The crystal structures and properties of the catalysts were analyzed by XRD, as shown in Figure 1. The XRD patterns of the CNS and PCNS samples contained similar diffraction peaks. Specifically, both contained characteristic peaks at 13.1° and 27.7°, which correspond to the (100) and (002) planes of g-C_3_N_4_, respectively (JCPDS 87-1526). The (100) plane represents the triazine ring structural unit of g-C_3_N_4_, while the (002) plane represents the layered arrangement of materials with graphite-like phases [30]. Thus, PCNS maintained a good g-C_3_N_4_ structure. No phosphorus peak was found in PCNS, probably because of the low phosphorus content. The peak intensities in the PCNS pattern were significantly weaker than those in the CNS pattern. Notably, this is indicative of reduced transport time for the electrons to travel from the interior to the surface of the material [26]. The characteristic peaks of BWO at 28.4°, 33.0°, 47.2°, 56.0°, 58.7°, 76.0°, and 78.5° correspond to the (131), (200), (202), (133), (262), (333), and (240) planes of Bi_2_WO_6_, respectively (JCPDS 73-1126) [31]. In addition, the diffraction peaks of the PCNS/BWO samples with different ratios were similar to those of BWO. As the content of PCNS increased, the intensity of the peak corresponding to the (002) plane of PCNS gradually increased, and the intensities of peaks corresponding to all planes of BWO gradually reduced, indicating that the PCNS/BWO composite catalysts were successfully prepared. 

**Figure 2:**
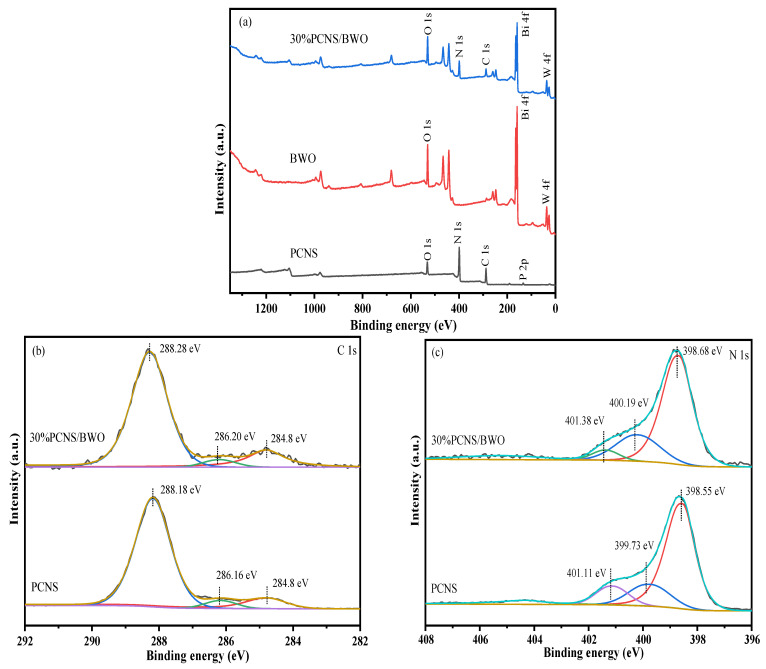

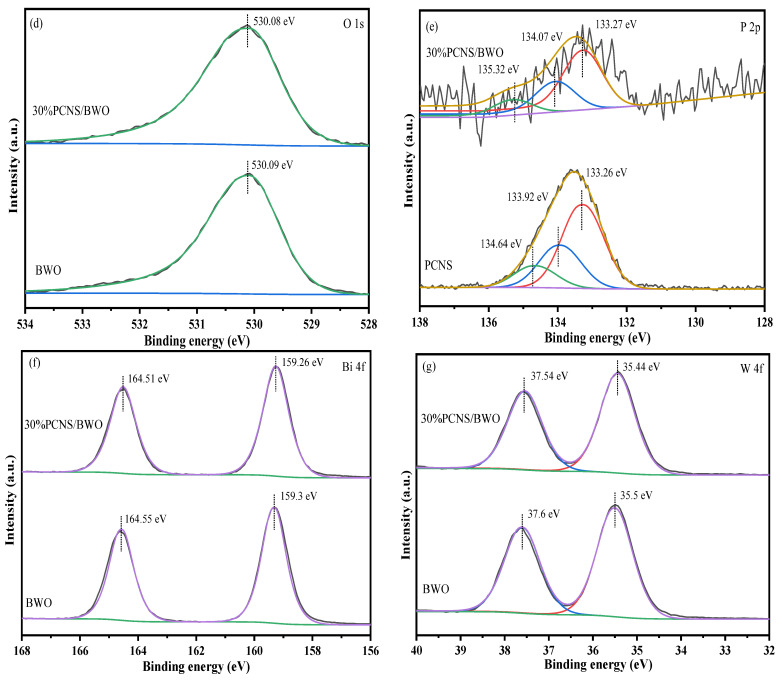
XPS data of PCNS, BWO, and 30% PCNS/BWO: (**a**) survey, (**b**) C 1s, (**c**) N 1s, (**d**) O 1s, (**e**) P 2p, (**f**) Bi 4f, and (**g**) W 4f spectra.

The surface compositions and valence states of the samples were analyzed by XPS. From the XPS survey spectra shown in Figure 2a, PCNS contained C, N, O, and P; BWO contained O, Bi, and W; and 30% PCNS/BWO contained C, N, O, Bi, and W. The absence of P in the 30% PCNS/BWO survey spectrum is probably due to the low phosphorus content. The C 1s spectrum of 30% PCNS/BWO (Figure 2b) contained peaks at 284.8, 286.2, and 288.28 eV, which correspond to C–C, C–N, and N–C=N bonds with sp^2^ hybridized C atoms, respectively [32]. The N 1s spectra (Figure 2c) contained peaks at 398.68, 400.19, and 401.38 eV, corresponding to C–N=C, N–(C)_3_, and C–NH_2_ bonds, respectively [33]. The O 1s spectra (Figure 2d) contained a single peak at 530.08 eV representing the Bi–O bond [34]. In the P 2p spectra (Figure 2e), three peaks at 133.27, 134.07, and 135.32 eV were observed, which correspond to P–N, P=N, and P=O bonds, respectively. This demonstrates that P–N and P=N covalent bonds form between the substitutional P atoms and adjacent N, and P=O bonds form between the P atoms and O from the air during the hydrothermal synthesis process [35]. These findings indicate that P was successfully doped into the CNS. The Bi 4f spectra (Figure 2f) contained Bi 4f_7/2_ and Bi 4f_5/2_ peaks at 159.26 and 164.51 eV, respectively, indicating the existence of Bi^3+^ [36], while the W 4f spectra (Figure 2 g) contained W 4f_7/2_ and W 4f_5/2_ peaks at 35.44 and 37.54 eV, respectively, indicating the existence of W^6+^ [11]. Importantly, the binding energies of the C 1s, N 1s, and P 2p peaks of 30% PCNS/BWO all shifted toward higher binding energies compared with those of PCNS, while the binding energies of the O 1s, Bi 4f, and W 4f peaks of 30% PCNS/BWO all shifted toward lower binding energies compared with those of BWO. Generally, if an element gains electrons, its binding energy decreases, and if it loses electrons, its binding energy increases [24]. These findings indicate that there was efficient electron transfer between PCNS and BWO, confirming the existence of a heterojunction structure.

**Figure 3:**
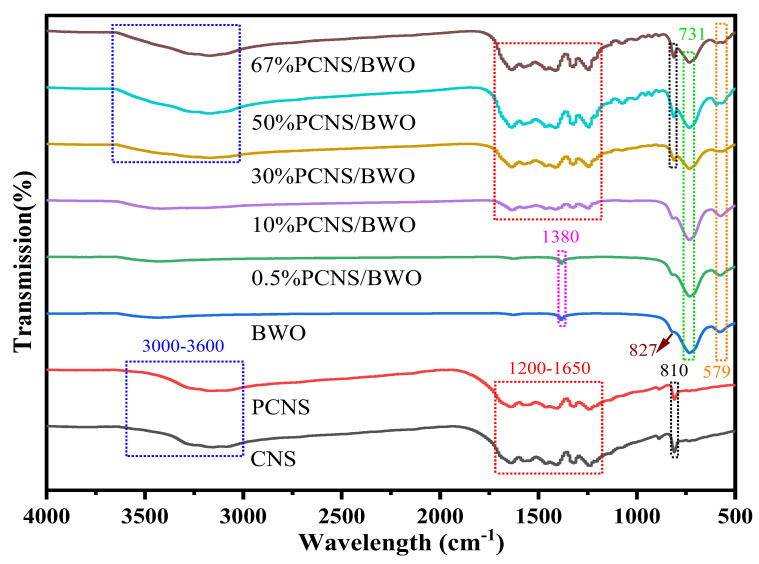
FTIR spectra of the synthesized samples.

The chemical structures of the samples were further analyzed by FTIR spectroscopy, as shown in Figure 3. The spectra of CNS and PCNS were very similar. The absorption bands at 3000–3600, 1200–1650, and 810 cm^−1^ correspond to N–H stretching vibrations, C–N heterocyclic stretching vibrations, and the triazine structure of g-C_3_N_4_ [37], respectively. This result confirms that P doping did not change the triazine ring structure of CNS, which is consistent with the XRD results. As the PCNS/BWO ratio increased, the intensities of all the peaks gradually increased. In the BWO spectrum, the absorption bands at 579, 731, 827, and 1380 cm^−1^ correspond to Bi–O–Bi stretching vibrations, W–O–W bonds, Bi–O stretching vibrations [38], and the vibration of the adsorbed water molecules [39], respectively. With increasing PCNS content, the peaks at 1380 and 827 cm^−1^ gradually disappeared, while those at 579 and 731 cm^−1^ gradually reduced. The characteristic peaks of PCNS and BWO coexisted in the spectra of the PCNS/BWO composites, indicating the successful construction of PCNS/BWO heterojunctions. 

**Figure 4:**
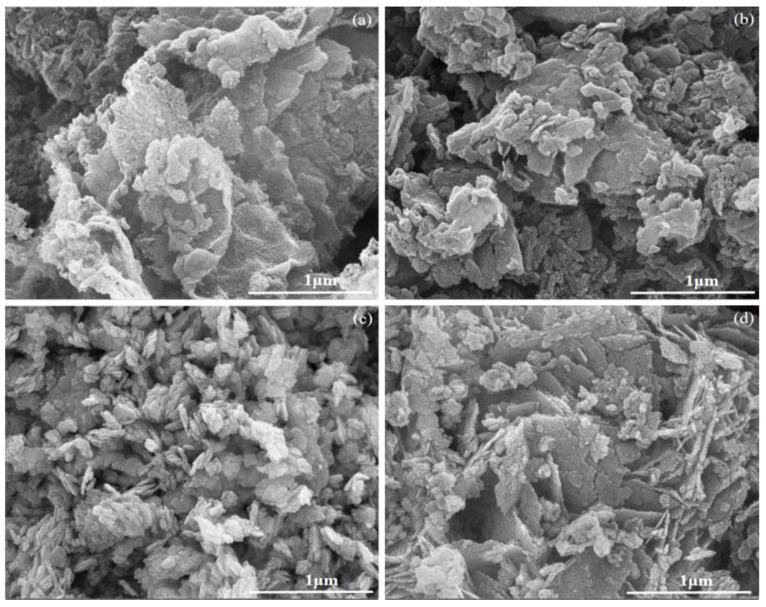
SEM images of (**a**) CNS, (**b**) PCNS, (**c**) BWO, and (**d**) 30% PCNS/BWO.

**Figure 5:**
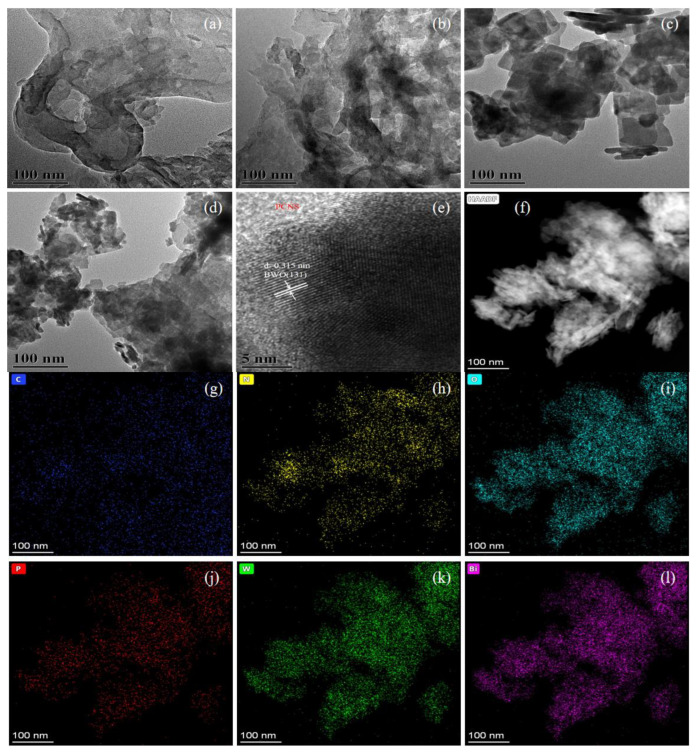
TEM images of (**a**) CNS, (**b**) PCNS, (**c**) BWO, and (**d**) 30% PCNS/BWO; (**e**) HRTEM image and (**f**) HAADF image of 30% PCNS/BWO; (**g**–**l**) EDS maps of the area in (**f**).

The morphologies and microstructures of the catalysts were characterized by SEM (Figure 4) and TEM (Figure 5). Figure 4a,b and Figure 5a,b demonstrate that the CNS and PCNS samples comprised large nanosheets with a 2D-layered structure. BWO was composed of smaller nanosheets with a 2D-layered structure, as shown in Figure 4c and Figure 5c. The 30% PCNS/BWO sample had a 2D/2D-layered structure, where the smaller BWO sheets were attached to the larger PCNS sheets (Figure 4d and Figure 5d). Figure 5e shows a high-resolution (HR)-TEM image of 30% PCNS/BWO. Clear lattice fringes can be seen, which were attributed to monolayer Bi_2_WO_6_ nanosheets (m-BWO), with a lattice spacing of 0.315 nm, corresponding to the (131) plane of orthorhombic Bi_2_WO_6_ [40]. The lattice at the edge of the image is PCNS. Notably, the interface between PCNS and BWO demonstrates that these components are in close contact, which is beneficial for the transfer of photogenerated carriers between them. In addition, from the energy-dispersive X-ray spectroscopy elemental mapping images in Figure 5g–l, it was confirmed that C, N, P, O, W, and Bi were uniformly distributed across the 30% PCNS/BWO sample, which further proves the successful preparation of the composite catalyst. The corresponding atomic composition is listed in Table S2. 

**Figure 6:**
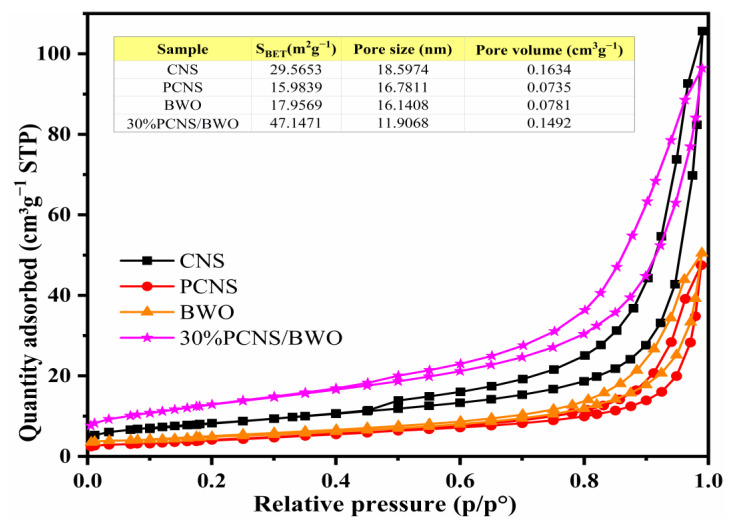
N_2_ adsorption/desorption isotherms of CNS, PCNS, BWO, and 30% PCNS/BWO.

The BET specific surface areas of the samples were calculated from the N_2_ adsorption/desorption isotherms shown in Figure 6. All of the samples had type IV isotherms with H3 type hysteresis, and the pore sizes were all less than 20 nm, indicating that the catalyst surfaces had mesoporous structures [41]. The specific surface areas of the CNS, PCNS, BWO, and 30% PCNS/BWO samples were 29.5653, 15.9839, 17.9569, and 47.1471 m^2^/g, respectively, and the relative pore volumes were 0.1634, 0.0735, 0.0781, and 0.1492 cm^3^/g, respectively. The specific surface area and relative pore volume of the 30% PCNS/BWO composite catalyst were significantly larger than those of PCNS and BWO. This indicates that the composite catalyst will exhibit better photocatalytic activity because the larger the specific surface area, the higher the adsorption capacity. Notably, these results are consistent with the photocatalytic activity measurements in Section 3.3. 

### 3.2. Optical Properties

**Figure 7:**
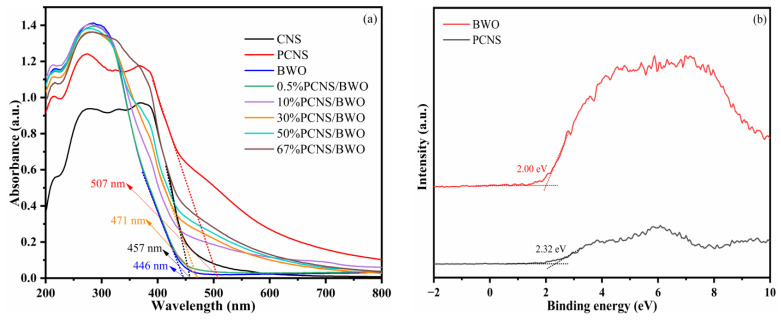
(**a**) UV-vis DRS spectra and (**b**) XPS-VB spectra of the catalysts.

To explore the light absorption behavior of the samples, UV-vis DRS was performed. The results are shown in Figure 7a. All of the catalysts were responsive to visible light, and the absorption edges of CNS, PCNS, BWO, and 30% PCNS/BWO were 457, 507, 446, and 471 nm, respectively. PCNS was more responsive to visible light than CNS, indicating that P doping effectively broadened the range of visible light absorption. The PCNS/BWO composite catalysts with different PCNS contents were all more responsive to visible light than BWO, indicating that the construction of 2D/2D PCNS/BWO heterojunctions prepared by combining morphological control and heterojunction construction effectively broadened the range of visible light absorption. The band gaps (*E*_g_) of the photocatalysts were calculated using Equation (1), from which the *E*_g_ values of PCNS and BWO were calculated to be 2.45 and 2.78 eV, respectively. The valence bands of PCNS and BWO were studied using XPS-VB spectroscopy, as shown in Figure 7b, and calculated to be 2.32 and 2.00 eV (relative to the Fermi level), respectively. The valence band edge potentials (*E*_VB_) relative to a normal hydrogen electrode (NHE) were calculated using Equation (2) [42], and the conduction band potentials (*E*_CB_) were calculated using Equation (3). The *E*_VB_ values of PCNS and BWO were calculated as 2.40 and 2.08 eV, respectively, and the *E*_CB_ values were calculated as −0.05 and −0.70 eV (vs. NHE, pH = 0), respectively.

*E*_g_ = 1240/*λ* (1)

*E*_VB_ = Ψ + VB_XPS_ − 4.44 (2)

*E*_CB_ = *E*_VB_ − *E*_g_ (3)

where *λ* is the absorption edge of the catalyst and Ψ is the electronic work function of the XPS instrument (4.52 eV).

**Figure 8:**
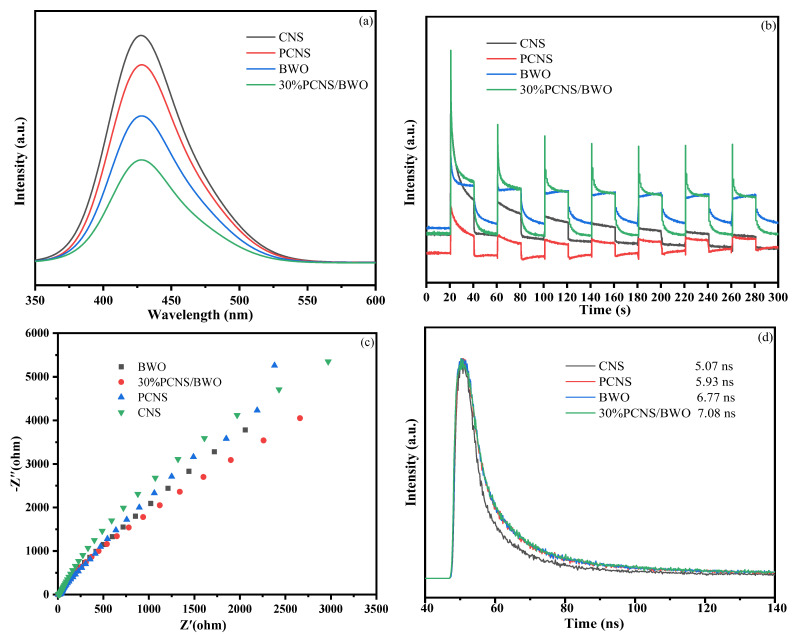
(**a**) Photoluminescence spectra, (**b**) *i*–*t* curves, (**c**) Nyquist plots from EIS data, and (**d**) fluorescence decay curves of CNS, PCNS, BWO, and 30% PCNS/BWO.

PL spectroscopy was performed at 428 nm to study the separation efficiencies of the photoinduced charge carriers in CNS, PCNS, BWO, and 30% PCNS/BWO. As shown in Figure 8a, CNS had the highest PL intensity, followed by PCNS, and 30% PCNS/BWO had the lowest PL intensity. Generally, a lower PL intensity corresponds to a higher electron–hole separation efficiency [43], indicating that 30% PCNS/BWO has the highest electron–hole separation efficiency. To further study the optoelectronic properties, photocurrent response (*i*–*t*) curves were constructed, and electrochemical impedance spectroscopy (EIS) was performed. As shown in Figure 8b,c, 30% PCNS/BWO had the largest photocurrent density and the smallest EIS arc radius, further confirming that 30% PCNS/BWO has the highest photogenerated electron–hole separation efficiency, which is one of the characteristics of excellent photocatalysts [44]. To further demonstrate the suppression of photogenerated electron–hole recombination, the carrier lifetimes in the reaction system were measured and analyzed. The time-resolved fluorescence spectra are shown in Figure 8d. The total lifetime of photogenerated charge carriers in 30% PCNS/BWO was 7.08 ns, which is larger than that for PCNS and BWO. A longer lifetime of photogenerated charge carriers means that they have more time to participate in the photocatalytic reaction, which is beneficial for improving photocatalytic efficiency [45]. From the discussion above, P doping effectively reduces the recombination rate of photoinduced electron–hole pairs, and the 2D/2D morphological control and heterojunction construction were also conducive to the effective separation of photoinduced electron–hole pairs, which was attributed to the optimized electronic band structure providing a more efficient electron transfer process [46]. 

### 3.3. Study of Photocatalytic Performance

**Figure 9:**
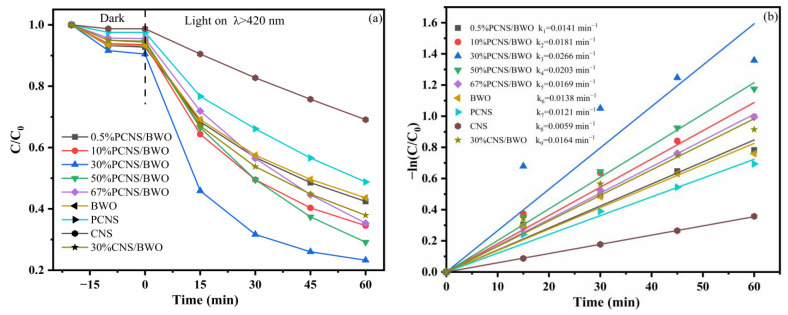
(**a**) Degradation curves of TC-HCl and (**b**) pseudo-first-order reaction kinetics of catalysis under visible light irradiation.

To evaluate the photocatalytic activities of the prepared samples, TC-HCl degradation experiments were performed in aqueous media under visible light irradiation. Currently, TC-HCl concentrations greater than 10 mg/L and catalyst concentrations greater than 0.2 g/L were used in similar published literature (see Table S3). In order to highlight the advanced nature of the catalysts used in this study, a higher concentration of TC-HCl (20 mg/L) and a lower concentration of catalyst (0.2 g/L) were chosen. It is expected to obtain the desired TC-HCl degradation rate with a higher concentration of TC-HCl and a lower concentration of catalyst. As shown in Figure 9a, under dark conditions, the reaction system reached adsorption–desorption equilibrium within 20 min. The PCNS/BWO composite catalysts had stronger adsorption capacities than PCNS, with 30% PCNS/BWO showing the highest adsorption capacity owing to its large specific surface area. The adsorption results were consistent with the BET analysis. After 60 min under visible light illumination, the degradation efficiency of PCNS was 51.2%, which was higher than that of CNS (30.9%), indicating that P doping significantly improved the photocatalytic efficiency. In the composite catalysts, as the content of PCNS was increased, the degradation efficiency of TC-HCl catalyzed by 2D/2D PCNS/BWO heterojunction gradually increased. However, when the PCNS content was too high, the separation of photogenerated carriers was inhibited, which gradually reduced the TC-HCl degradation efficiency. Nonetheless, the degradation efficiencies of all the composite catalysts were higher than those of BWO and PCNS, with 30% PCNS/BWO exhibiting the highest degradation efficiency of 76.7%. This corresponds to a significant improvement compared with that of BWO and PCNS owing to the optimized energy band structure and optical properties of the composite catalyst. For comparison, the degradation efficiency of the 30% CNS/BWO (without P) was 62.1%, confirming that P doping improved the photocatalytic efficiency.

The kinetic rate constants of TC-HCl degradation under visible light irradiation are shown in Figure 9b. All kinetic equations were linear, indicating that the degradations followed the pseudo-first-order kinetic model. The rate constants of degradations catalyzed by CNS, PCNS, BWO, and 30% PCNS/BWO were 0.0059, 0.0121, 0.0138, and 0.0266 min^−1^, respectively. The degradation rate constant of the 30% PCNS/BWO catalyst was 4.5 times greater than that of the CNS catalyst and effectively improved compared with those of PCNS and BWO. These results suggest that P doping and the construction of 2D/2D heterojunctions are effective approaches to enhance photocatalytic performance. In addition, the measured TC-HCl degradation efficiency of 30% PCNS/BWO is higher than the reported values of most BWO-based photocatalysts (Table S3), indicating that 30% PCNS/BWO is a good photocatalyst for the removal of organic pollutants. 

**Figure 10:**
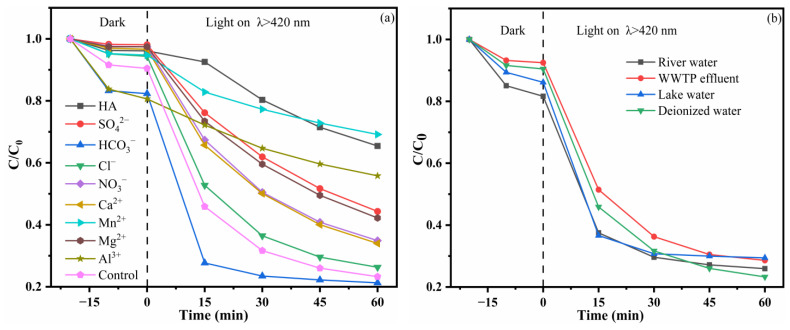
TC-HCl degradation catalyzed by 30% PCNS/BWO (**a**) with humic acid (10 mg/L) and different anions and cations (5 mM), and (**b**) with different water sources (20 mg/L).

To evaluate the suitability of the 30% PCNS/BWO composite photocatalyst for practical applications, the effects of humic acid, different anions and cations (HCO_3_^−^, Cl^−^, NO_3_^−^, SO_4_^2−^, Ca^2+^, Mg^2+^, Al^3+^, Mn^2+^), and different water sources (river water, effluent of the sewage treatment plant, lake water, deionized water) on the photocatalytic degradation efficiency of TC-HCl were studied. The results of humic acid and different anions and cations are shown in Figure 10a. Humic acid severely inhibited the TC-HCl degradation ability. Humic acid contains chromophores such as benzene rings, carboxyl groups, and carbonyl groups that compete with the catalyst for light, which inhibits photodegradation [47]. For the anions, HCO_3_^−^ promoted the degradation of TC-HCl. This is reasonable considering that HCO_3_^−^ could react with •OH to generate •CO_3_^−^, that •OH was not the main active species in the reaction system (see Section 3.4), and that •CO_3_^−^ could accelerate the degradation of TC-HCl [48]. Cl^−^ had little effect on the degradation of TC-HCl, while NO_3_^−^ and SO_4_^2−^ significantly inhibited the degradation of TC-HCl. This might be because the anionic charges compete with pollutants for active sites on the catalyst surface, thereby inhibiting the degradation of TC-HCl [49]. The cations, including Ca^2+^, Mg^2+^, Al^3+^, and Mn^2+^, all seriously inhibited the degradation of TC-HCl because they can combine with TC-HCl to form metal complexes, thus reducing the rate of degradation [50]. The results of using different water sources are shown in Figure 10b. When using river water, the effluent of a sewage treatment plant, and lake water, the TC-HCl degradation was slightly inhibited compared with that in deionized water. This might be caused by dissolved organic matter and ions in these water sources. However, the degradation ability remained high. This indicates that the 30% PCNS/BWO photocatalyst can be used in the practical treatment of TC-HCl-containing wastewater, which is promising for industrial applications.

**Figure 11:**
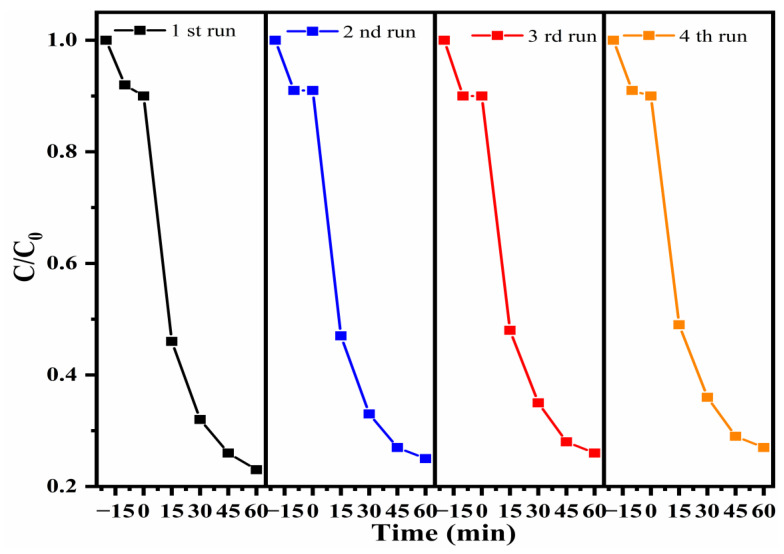
Cycling experiment of TC-HCl degradation catalyzed by 30% PCNS/BWO.

**Figure 12:**
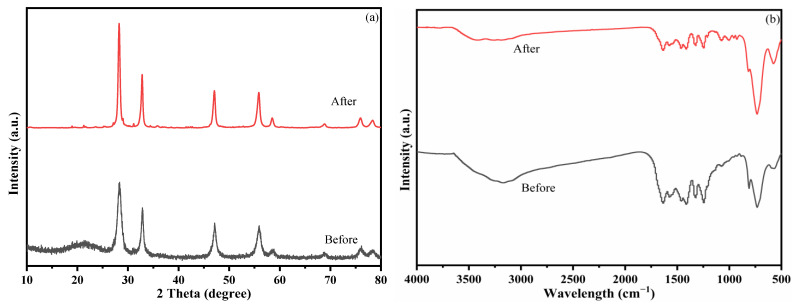
(**a**) XRD patterns and (**b**) FTIR spectra of 30% PCNS/BWO before and after cycling experiment.

The reusability and stability of photocatalysts are important for industrial applications. To evaluate this, the same 30% PCNS/BWO composite was used in four repeat TC-HCl degradation experiments, and the catalyst was characterized by XRD and FTIR before the first run and after the fourth run. As shown in Figure 11, the rate of TC-HCl degradation decreased slightly after four runs but still reached 73%, indicating that the photocatalyst had high stability and good performance. As shown in Figure 12a,b, the XRD patterns and FTIR spectra of the 30% PCNS/BWO composite after cycling were almost the same as those of the pristine catalyst, indicating that the structure and chemical surface properties of the composite photocatalyst remained stable. 

### 3.4. Mechanism and Pathway of Degradation

**Figure 13:**
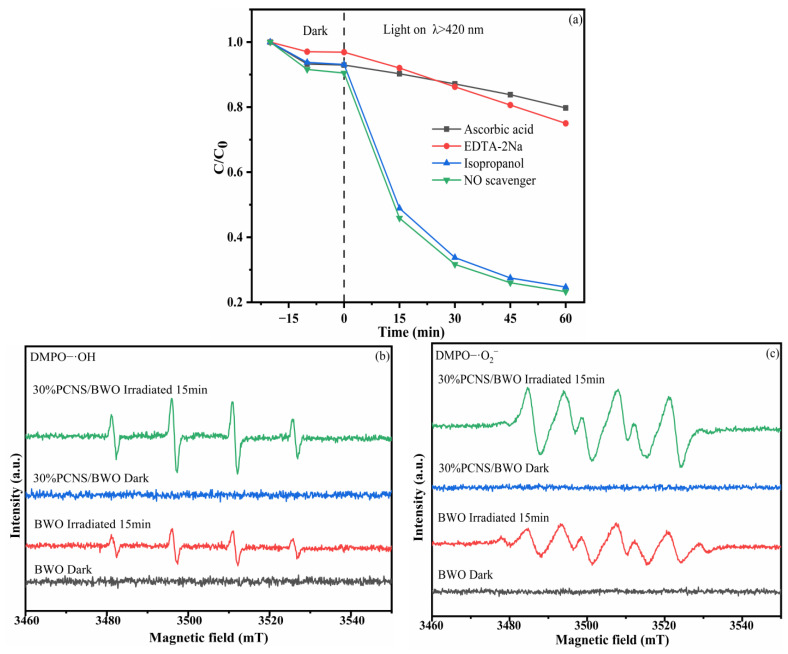
(**a**) Effects of different scavengers on the degradation of TC-HCl catalyzed by 30% PCNS/BWO; ESR spectra of (**b**) DMPO-•OH and (**c**) DMPO-•O_2_^−^.

Free radicals play a major role in photocatalytic degradation. Here, scavenging experiments were performed to analyze the active species in the photocatalytic degradation of TC-HCl catalyzed by the 30% PCNS/BWO composite. Ascorbic acid, EDTA-2Na, and IPA were used to scavenge •O_2_^−^, h^+^, and •OH, respectively [51,52]. The results are shown in Figure 13a. When ascorbic acid was added to the reaction system, the photocatalytic process was greatly inhibited, and the removal rate of TC-HCl decreased from 76.7% to 20.3%, indicating that •O_2_^−^ has a great influence on the degradation of TC-HCl. Similarly, the TC-HCl degradation ability was greatly suppressed after adding EDTA-2Na, with a removal rate of just 25.0%, indicating that h^+^ also plays a crucial role in the degradation of TC-HCl. In contrast, when IPA was added, the removal rate of TC-HCl decreased only slightly (75.3%), which indicates that while •OH participates in the photodegradation, it is not the main active species. These results indicate that •O_2_^−^ and h^+^ are the main active species in the photocatalytic reaction, while •OH plays a participating role.

To further confirm the participation of free radicals in the photocatalytic degradation of TC-HCl, ESR spectroscopy was performed by adding DMPO to the reaction system, and the results are shown in Figure 13b,c. Neither 30% PCNS/BWO nor BWO systems produced DMPO-•OH or DMPO-•O_2_^−^ signals under dark conditions, but both produced obvious DMPO-•OH and DMPO-•O_2_^−^ signals after 15 min of visible light illumination, indicating that •OH and •O_2_^−^ were generated by a photocatalytic process. The signal intensities in the 30% PCNS/BWO spectrum were higher than those in the BWO spectrum, which indicates that the 30% PCNS/BWO composite had a stronger oxidizing ability than BWO.

**Figure 14:**
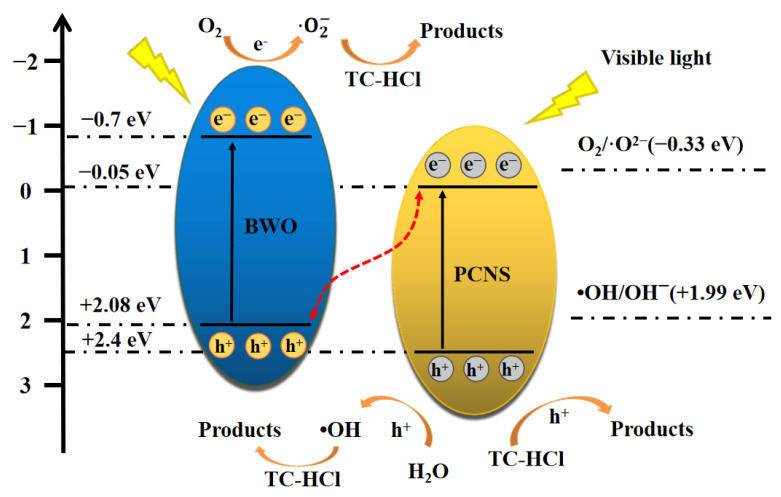
Mechanism of TC-HCl degradation catalyzed by 30% PCNS/BWO.

The mechanism of photocatalytic TC-HCl degradation using the 30% PCNS/BWO composite catalyst under visible light irradiation was proposed based on the above analysis, as shown in Figure 14. The *E*_VB_ values of PCNS and BWO were 2.4 and 2.08 eV, respectively, and the *E*_CB_ values were −0.05 and −0.7 eV (vs. NHE, pH = 0), respectively (Figure 7). Thus, both PCNS and BWO are excited by visible light irradiation. The photogenerated electrons transfer from the valence band to the conduction band while the photogenerated holes remain on the valence band. 

The transfer of electrons and holes between PCNS and BWO might follow type-II or direct Z-scheme heterojunction behavior. If type-II heterojunction behavior occurs, then the electrons would transfer from the conduction band of BWO to that of PCNS, while the holes would transfer from the valence band of PCNS to that of BWO. However, since the *E*_CB_ of PCNS (−0.05 eV) is more positive than the standard redox potential of O_2_/•O_2_^−^ (−0.33 eV vs. NHE) [53], the reaction between e^−^ in the conduction band of PCNS and O_2_ would not produce •O_2_^−^, which contradicts the findings of the scavenging experiments and ESR spectroscopy. Thus, the transfer of electrons and holes between PCNS and BWO does not follow type-II heterojunction behavior. If direct Z-scheme heterojunction behavior occurs, then the electrons in the conduction band of PCNS would transfer to the valence band of BWO, which would effectively reduce the electron–hole pair recombination rate and improve the photocatalytic activity. As the *E*_CB_ of BWO (−0.7 eV) is more negative than the standard redox potential of O_2_/•O_2_^−^ (−0.33 eV vs. NHE) [53], the reaction between e^−^ in the conduction band of BWO and O_2_ could produce •O_2_^−^. Furthermore, as the *E*_CB_ of PCNS (+2.4 eV) is more positive than the standard redox potential of •OH/OH^−^ (+1.99 eV vs. NHE) [54], the reaction between h^+^ and H_2_O or OH^−^ could produce •OH. These findings are consistent with the scavenging experiments and ESR spectroscopy, which confirms that the transfer of electrons and holes between PCNS and BWO occurs by direct Z-scheme heterojunction behavior. 

The active species generated during the photocatalytic process (i.e., h^+^, •OH in the valence band of PCNS, and •O_2_^−^ in the conduction band of BWO) then oxidized and decomposed TC-HCl into intermediates, CO_2_, and H_2_O, respectively. The possible reactions are described in Equations (4)–(8):

PCNS + hν → PCNS(e^−^ + h^+^) (4)

BWO + hν → BWO(e^−^ + h^+^) (5)

BWO(e^−^) + O_2_ → •O_2_^−^ (6)

PCNS(h^+^) + H_2_O/OH^−^ → •OH (7)

PCNS(h^+^ + •OH) + BWO(•O_2_^−^) + TC-HCl → intermediates + CO_2_ + H_2_O (8)

**Figure 15:**
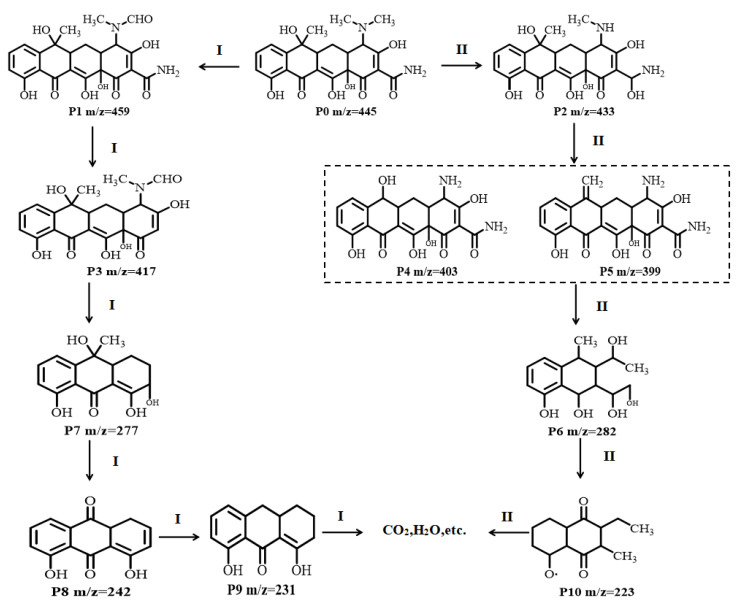
Possible pathways of TC-HCl degradation catalyzed by 30% PCNS/BWO.

To further explore the degradation of TC-HCl, HRAM LC-MS/MS was used to detect the degradation intermediates after 60 min of visible light illumination. Because H^+^ and Cl^−^ from TC-HCl exist in the system as free ions, only P0 (*m*/*z* = 445) could be photocatalyzed and detected. A total of 10 intermediates were detected during the degradation (Figure S1, *m*/*z* values of 459, 433, 417, 403, 399, 282, 277, 242, 231, and 223). Two possible degradation pathways were proposed (Figure 15). In degradation pathway I, –CH_3_ on P0 (*m*/*z* = 445) is oxidized to –CHO by the active species generated during the photocatalytic process to produce P1 (*m*/*z* = 459) [55]. P1 is then deamidated to produce P3 (*m*/*z* = 417) [56]. Through the ring opening of benzene, P3 becomes P7 (*m*/*z* = 277) [57]. Through demethylation and dehydroxylation, P7 becomes P8 (*m*/*z* = 242) and then P9 (*m*/*z* = 231) [58]. In degradation pathway II, P0 becomes P2 (*m*/*z* = 433) through demethylation [59]. P2 then becomes P4 (*m*/*z* = 403) and P5 (*m*/*z* = 399) through demethylation and oxidation by hydroxyl and active species, followed by ring opening to produce P6 (*m*/*z* = 282) [60]. Through various processes, including the loss of C=C bonds, carbonylation, and dehydroxylation, P6 becomes P10 (*m*/*z* = 223) [61]. Finally, all these intermediates are oxidized to substances such as CO_2_ and H_2_O.

## 4. Conclusions

In conclusion, 2D/2D P-doped g-C_3_N_4_/Bi_2_WO_6_ direct Z-scheme heterojunction catalysts were prepared using a hydrothermal method, with 2D Bi_2_WO_6_ nanosheets loaded on 2D P-doped g-C_3_N_4_ nanosheets. The efficiency of the synthesized 30% P-doped g-C_3_N_4_/Bi_2_WO_6_ catalyst for the photocatalytic degradation of tetracycline hydrochloride under visible light irradiation was much higher than that of P-doped g-C_3_N_4_ and Bi_2_WO_6_. Through doping, P atoms replaced C atoms in the g-C_3_N_4_ nanosheets and covalently bonded to the adjacent N atoms, which effectively broadened the range of visible light absorption of g-C_3_N_4_. The heterojunction constructed between 2D P-doped g-C_3_N_4_ and 2D Bi_2_WO_6_ increased the specific surface area, and the close contact at the interface accelerated the transfer of photogenerated charge carriers, reduced the recombination rate of photoinduced electrons and holes, increased the lifetimes of the charge carriers, and optimized the electronic band structure. Therefore, it synergistically improved the photocatalytic performance along with P doping. •O_2_^−^ and h^+^ were the main active species in the photocatalytic reaction system, and •OH also participated in the process. Using high-resolution accurate mass liquid chromatography with tandem mass spectrometry, it was found that tetracycline hydrochloride produced a total of 10 degradation intermediates in the photocatalytic process, and two degradation pathways were proposed, mainly involving the processes of deamidation, ring opening of benzene, demethylation, and dehydroxylation. Importantly, the degradation of tetracycline hydrochloride was only slightly inhibited when using different water sources, including river water, the effluent of a sewage treatment plant, and lake water, which indicates that the 30% P-doped g-C_3_N_4_/Bi_2_WO_6_ photocatalyst has great application potential for the treatment of sewage. This work provides insight into the preparation of high-performance photocatalysts by combining multiple strategies simultaneously, including morphological control, doping, and heterojunction construction. 

## Data Availability

Not applicable.

## Supplementary Materials

The following supporting information can be downloaded at: https://www.mdpi.com/article/10.3390/ijerph192214935/s1, Table S1. Feedstock dosage for each catalyst. Table S2. Atomic percentage of different elements present in 30%PCNS/BWO samples. Table S3. Comparison with other BWO-based photocatalysts for the degradation of TC-HCl [31,38,62,63,64,65,66,67,68,69,70]. Figure S1. HRAMLC-MS/MS secondary ion mass spectra of TC-HCl photocatalytic degradation products.
