# Supplementary material for: 2D/2D Phosphorus-Doped g-C3N4/Bi2WO6 Direct Z-Scheme Heterojunction Photocatalytic System for Tetracycline Hydrochloride (TC-HCl) Degradation
Source: Int J Environ Res Public Health. 2022 Nov 13;19(22):14935. doi: 10.3390/ijerph192214935 (PMC9691143; doi:10.3390/ijerph192214935)
Supplement: Supplementary file 1 [file ijerph-19-14935-s001.zip › ijerph-2005181-supplementary.pdf]

Supplementary Material

2D/2D Phosphorus-Doped g-C_3N_4/Bi_2WO_6 Direct Z-Scheme Heterojunction Photocatalytic System for Tetracycline Hydrochloride (TC-HCl) Degradation

Xudong Yin[1,2], Xiaojie Sun[1,*], Dehao Li[2,*], Wenyu Xie[2], Yufeng Mao[2], Zhenghui Liu[2], Zhisen Liu[2]

1 Guangxi Key Laboratory of Environmental Pollution Control Theory and Technology, College of Environmental Science and Engineering, Guilin University of Technology, Guilin 541006, China
2 Guangdong Provincial Key Laboratory of Petrochemical Pollution Processes and Control, Key Laboratory of Petrochemical Pollution Control of Guangdong Higher Education Institutes, School of Environmental Science and Engineering, Guangdong University of Petrochemical Technology, Maoming 525000, China

* Correspondence:
sunxiaojie@glut.edu.cn (X.S.)
ldh@gdupt.edu.cn (D.L.)

Table S1. Feedstock dosage for each catalyst

Photocatalysts	PCNS (g)	CNS (g)	$Bi(NO_3)_3·5H_2O$ (g)	$Na_2WO_4·2H_2O$ (g)
0.5%PCNS/BWO	0.0035	0	0.9701	0.3299
10%PCNS/BWO	0.0698	0	0.9701	0.3299
30%PCNS/BWO	0.2093	0	0.9701	0.3299
50%PCNS/BWO	0.3489	0	0.9701	0.3299
67%PCNS/BWO	0.4675	0	0.9701	0.3299
BWO	0	0	0.9701	0.3299
30%CNS/BWO	0	0.2093	0.9701	0.3299

Table S2. Atomic percentage of different elements present in 30%PCNS/BWO samples

	C	N	O	P	W	Bi
At%	26.33	6.45	47.84	0.90	6.75	11.73

Table S3. Comparison with other BWO-based photocatalysts for the degradation of TC-HCl

Photocatalysts	TC-HCl Concentration (mg/L)	Dosage (g/L)	Time (min)	Light source	Removal (%)	Kinetic Constant (min^{-1})	References
$Co_3O_4/Ag/Bi_2WO_6$	10	1.0	60	300 W xenon lamp (λ > 420 nm)	57.2	0.0157	[1]
7-MIL-88B(Fe)/Bi_2WO_6	10	0.5	90	500 W xenon lamp (λ > 420 nm)	96.4	0.0268	[2]
5%MIL-125(Ti)/ Bi_2WO_6	20	0.4	80	300 W xenon lamp (λ > 400 nm)	73.0	0.0184	[3]
BiOCl/Bi_2WO_6-2	20	0.7	150	300 W xenon lamp (λ > 420 nm)	63.9	0.0067	[4]
Bi_2WO_6/BiOBr	10	0.2	90	300 W xenon lamp (λ > 420 nm)	62.2	/	[5]
Bi_2WO_6/Nb_2CT_x	15	0.5	120	500 W xenon lamp (λ > 420 nm)	83.1	0.0171	[6]
15%$CuInS_2/Bi_2WO_6$	10	0.3	120	300 W xenon lamp (λ > 420 nm)	92.4	0.0176	[7]
Bi_2WO_6/Ta_3N_5	20	0.4	120	300 W xenon lamp (λ > 400 nm)	86.7	0.0169	[8]
g-C_3N_4/Bi_2WO_6	10	0.4	60	300 W xenon lamp (λ > 420 nm)	≈65.0	0.0177	[9]
g-C_3N_4/Bi_2WO_6	10	1.0	120	300 W xenon lamp (λ > 420 nm)	≈64.0	/	[10]
g-C_3N_4/Bi_2WO_6	20	0.6	60	300 W xenon lamp (λ > 420 nm)	76.25	0.02	[11]
30%CNS/BWO	20	0.2	60	300 W xenon lamp (λ > 420 nm)	62.1	0.0164	This work
30%PCNS/BWO	20	0.2	60	300 W xenon lamp (λ > 420 nm)	76.7	0.0266	This work

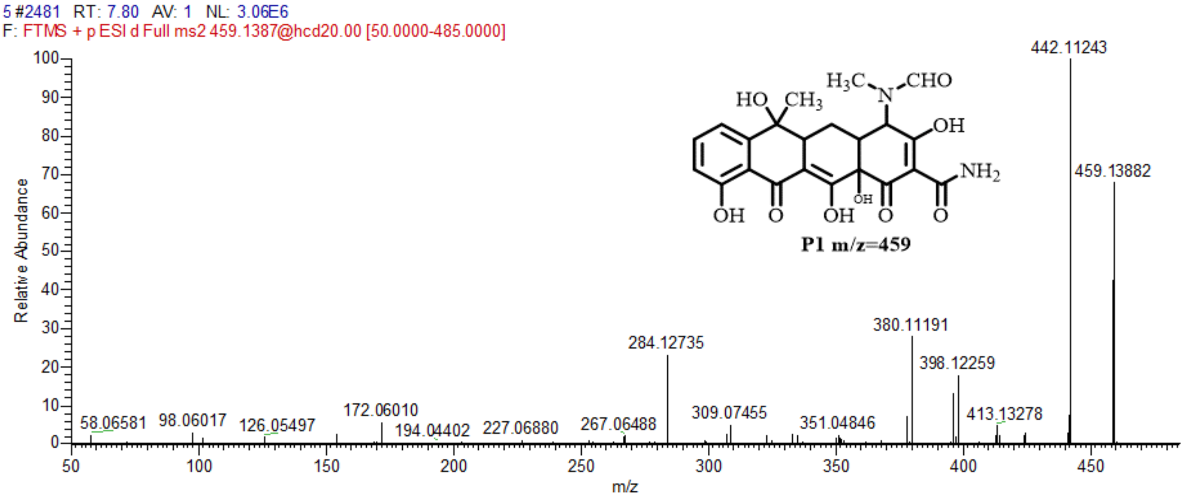
5 #2481 RT: 7.80 AV: 1 NL: 3.06E6
F: FTMS + p ESI d Full ms2 459.1387@hcd20.00 [50.0000-485.0000]
Relative Abundance
m/z
442.11243
459.13882
380.11191
398.12259
284.12735
413.13278
309.07455
351.04846
267.06488
227.06880
194.04402
172.06010
126.05497
98.06017
58.06581
P1 m/z=459

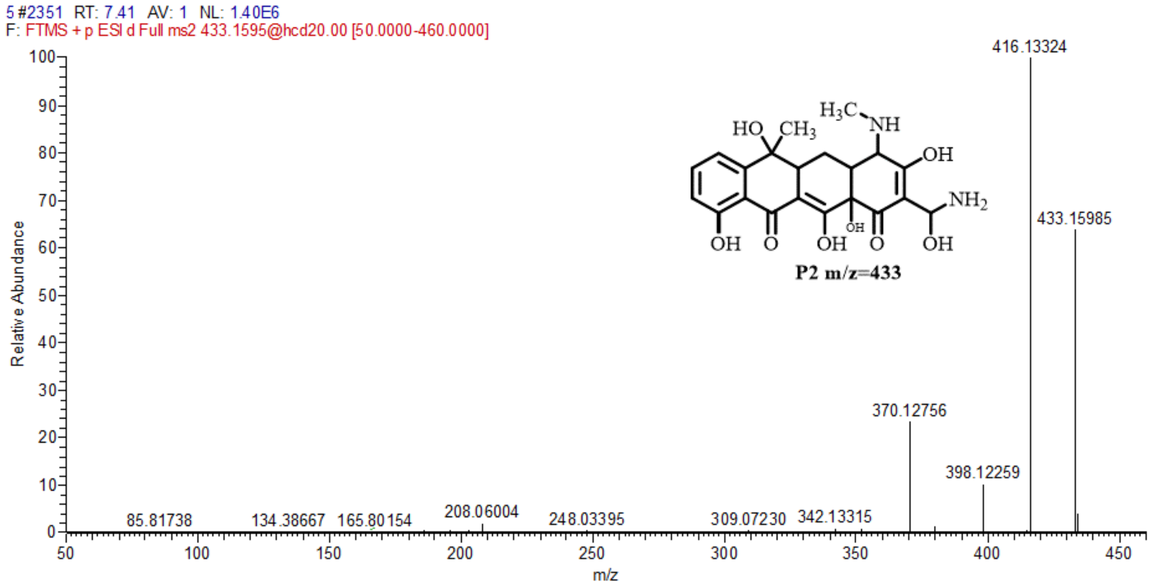
5 #2351 RT: 7.41 AV: 1 NL: 1.40E6
F: FTMS + p ESI d Full ms2 433.1595@hcd20.00 [50.0000-460.0000]
Relative Abundance
m/z
416.13324
433.15985
370.12756
398.12259
85.81738
134.38667
165.80154
208.06004
248.03395
309.07230
342.13315
P2 m/z=433

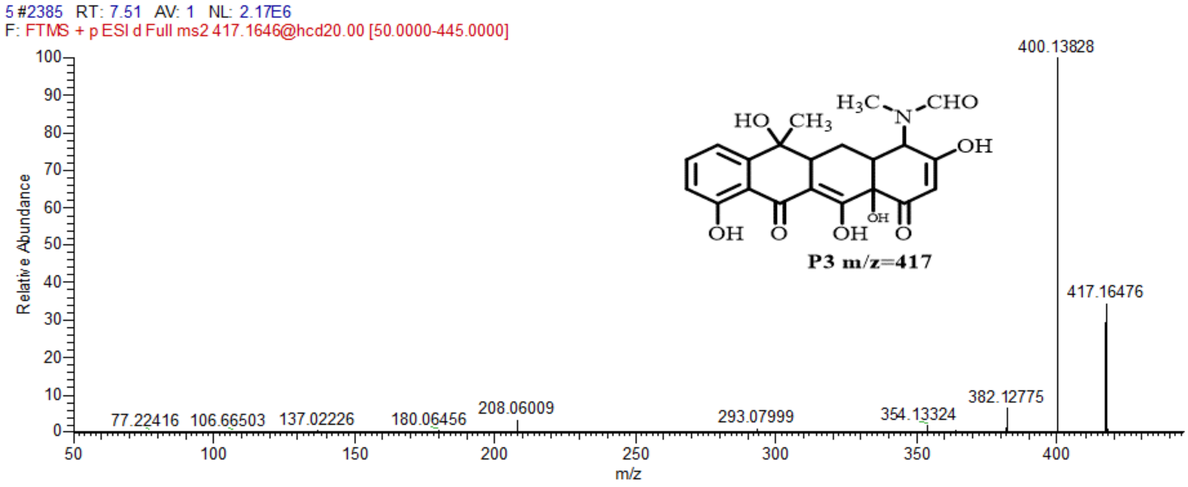
5 #2385 RT: 7.51 AV: 1 NL: 2.17E6
F: FTMS + p ESI d Full ms2 417.1646@hcd20.00 [50.0000-445.0000]
Relative Abundance
m/z
400.13828
417.16476
382.12775
354.13324
293.07999
208.06009
180.06456
137.02226
106.66503
77.22416
P3 m/z=417

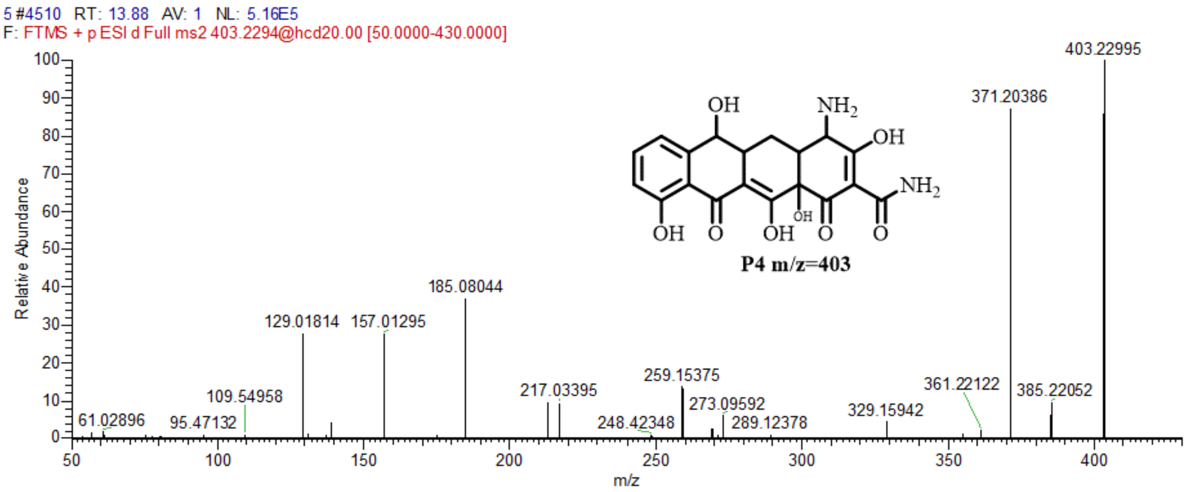
5 #4510 RT: 13.88 AV: 1 NL: 5.16E5
F: FTMS + p ESI d Full ms2 403.2294@hcd20.00 [50.0000-430.0000]
Relative Abundance
m/z
403.22995
371.20386
185.08044
129.01814
157.01295
109.54958
61.02896
95.47132
217.03395
259.15375
273.09592
248.42348
289.12378
329.15942
361.22122
385.22052
P4 m/z=403

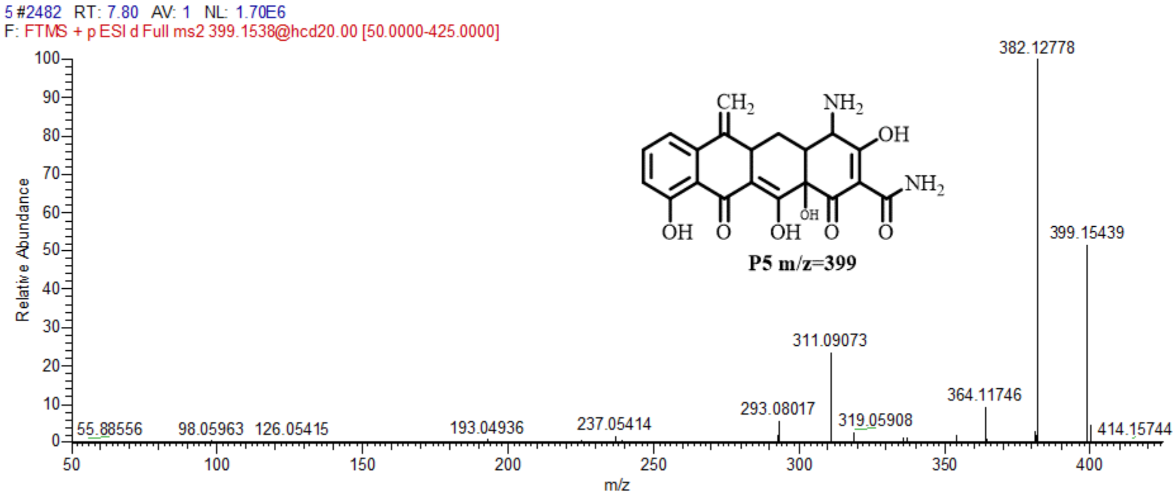
5 #2482 RT: 7.80 AV: 1 NL: 1.70E6
F: FTMS + p ESI d Full ms2 399.1538@hcd20.00 [50.0000-425.0000]
Relative Abundance
m/z
382.12778
399.15439
311.09073
364.11746
293.08017
319.05908
237.05414
193.04936
55.88556
98.05963
126.05415
414.15744
P5 m/z=399

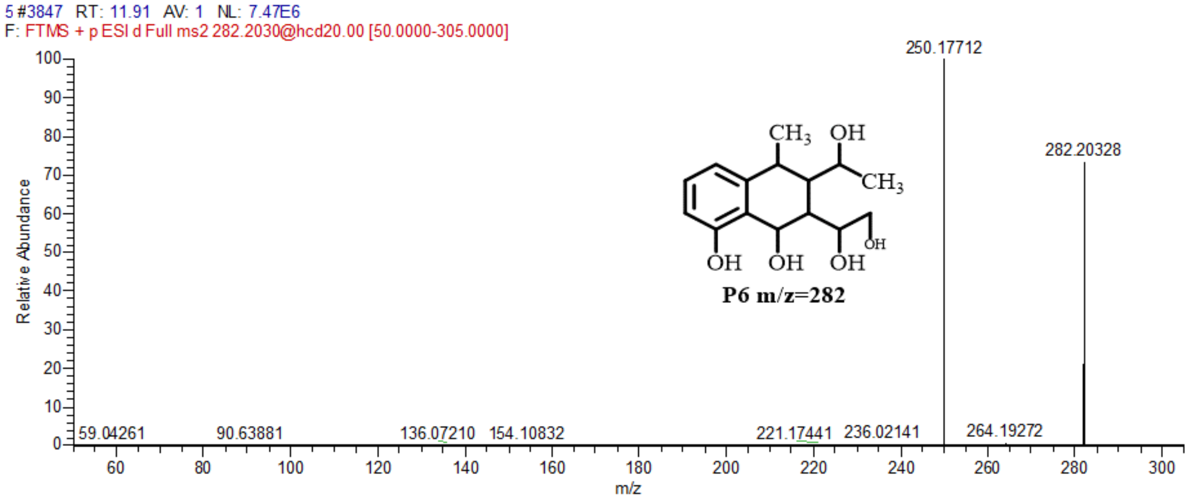
5 #3847 RT: 11.91 AV: 1 NL: 7.47E6
F: FTMS + p ESI d Full ms2 282.2030@hcd20.00 [50.0000-305.0000]
Relative Abundance
m/z
250.17712
282.20328
59.04261
90.63881
136.07210
154.10832
221.17441
236.02141
264.19272
P6 m/z=282

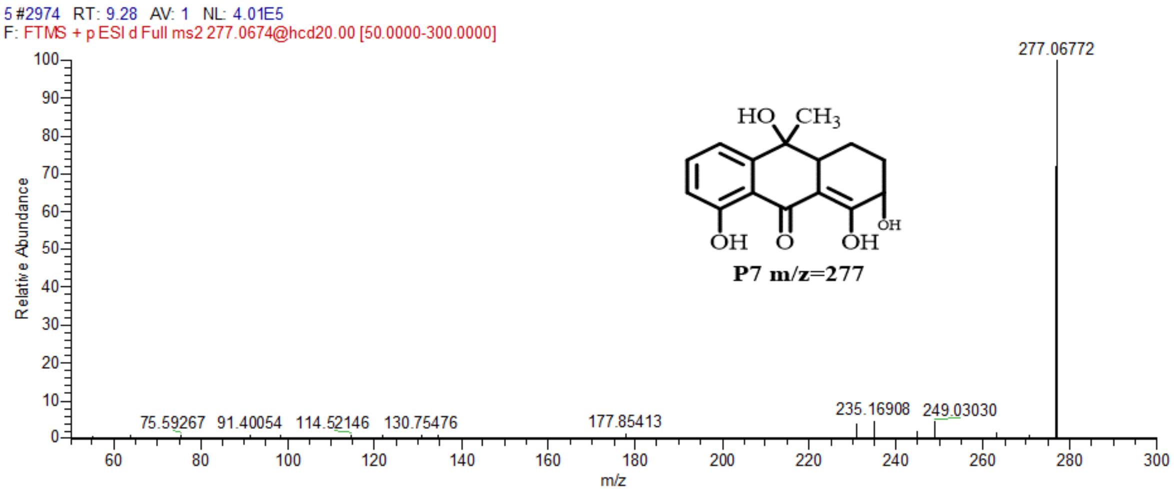
5 #2974 RT: 9.28 AV: 1 NL: 4.01E5
F: FTMS + p ESI d Full ms2 277.0674@hcd20.00 [50.0000-300.0000]
277.06772
75.59267 91.40054 114.52146 130.75476
177.85413
235.16908 249.03030
Relative Abundance
m/z
HO CH3
OH O OH
OH
P7 m/z=277

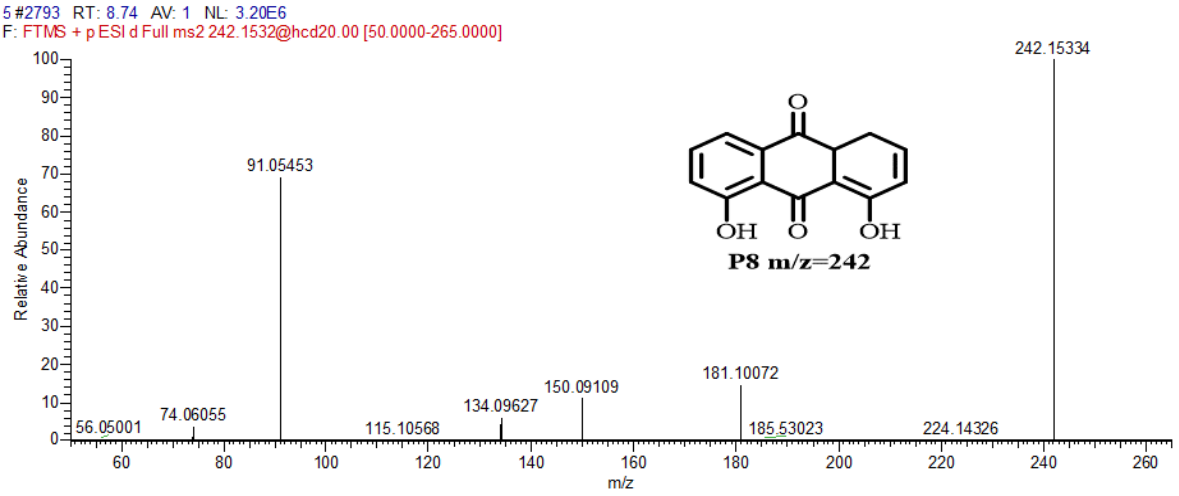
5 #2793 RT: 8.74 AV: 1 NL: 3.20E6
F: FTMS + p ESI d Full ms2 242.1532@hcd20.00 [50.0000-265.0000]
242.15334
91.05453
56.05001
74.06055
115.10568
134.09627
150.09109
181.10072
185.53023
224.14326
Relative Abundance
m/z
O
OH O OH
P8 m/z=242

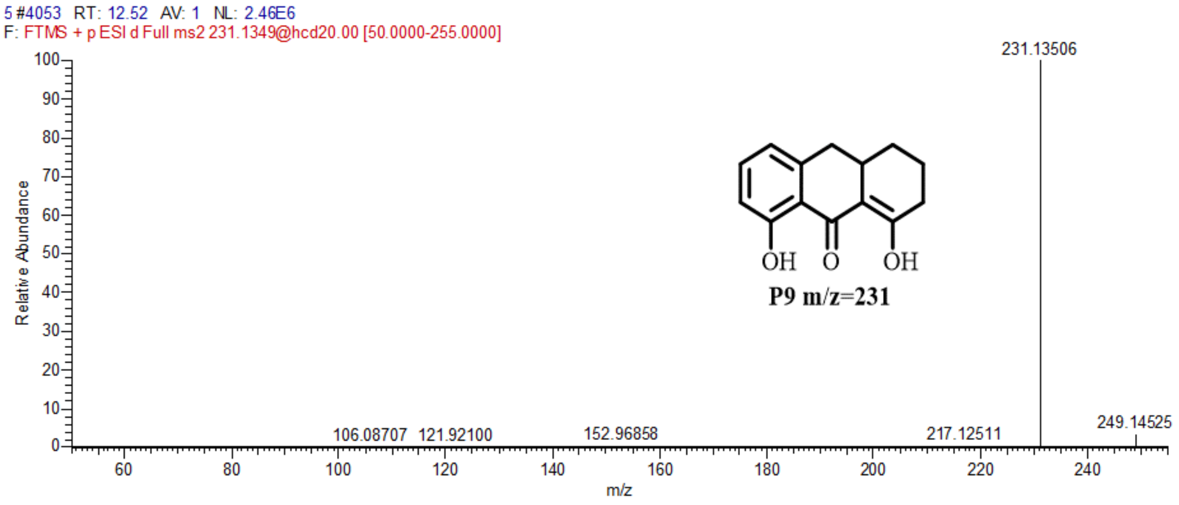
5 #4053 RT: 12.52 AV: 1 NL: 2.46E6
F: FTMS + p ESI d Full ms2 231.1349@hcd20.00 [50.0000-255.0000]
231.13506
106.08707 121.92100
152.96858
217.12511
249.14525
Relative Abundance
m/z
OH O OH
P9 m/z=231

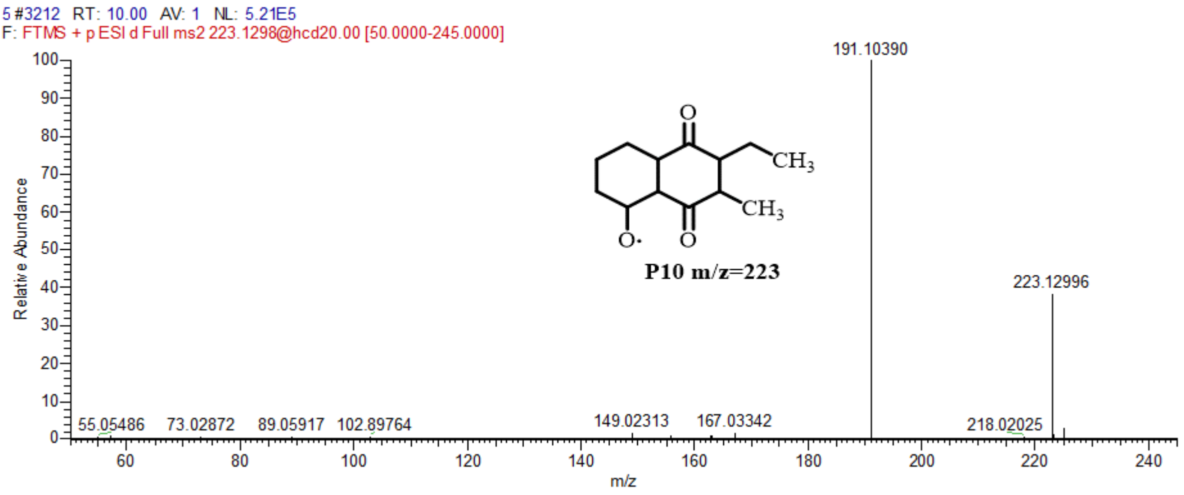

Figure S1. HRAMLC-MS/MS secondary ion mass spectra of TC-HCl photocatalytic degradation products

References

1.Wan, J.; Xue, P.; Wang, R.M.; Liu, L.; Liu, E.Z.; Bai, X.; Fan, J.; Hu, X.Y. Synergistic effects in simultaneous photocatalytic removal of Cr (VI) and tetracycline hydrochloride by Z-scheme Co_3O_4/Ag/Bi_2WO_6 heterojunction. *Appl. Surf. Sci.* **2019,** *483*, 677-687.

2.Hu, P.B.; Yao, C.F.; Yang, L.; Xin, Y.M.; Miao, Y.Q. Boosted photodegradation of tetracycline hydrochloride over Z-scheme MIL-88B (Fe)/Bi_2WO_6 composites under visible light. *Colloids Surf. Physicochem. Eng. Aspects* **2021,** *627*, 127248.

3.Yin, S.; Chen, Y.; Gao, C.; Hu, Q.S.; Li, M.; Ding, Y.; Di, J.; Xia, J.X.; Li, H.M. In-situ preparation of MIL-125(Ti)/Bi_2WO_6 photocatalyst with accelerating charge carriers for the photodegradation of tetracycline hydrochloride. *J. Photochem. Photobiol. A: Chem.* **2020,** *387*, 112149.

4.Peng, R.F.; Kang, Y.X.; Deng, X.H.; Zhang, X.M.; Xie, F.; Wang, H.Y.; Liu, W.W., Topotactic transformed face-to-face heterojunction of BiOCl/Bi_2WO_6 for improved tetracycline photodegradation. *J. Environ. Chem. Eng.* **2021,** *9*, 106750.

5.Ren, X.Z.; Wu, K.; Qin, Z.G.; Zhao, X.C.; Yang, H. The construction of type II heterojunction of Bi_2WO_6/BiOBr photocatalyst with improved photocatalytic performance. *J. Alloy. Compd.* **2019,** *788*, 102-109.

6.Cui, C.; Guo, R.H.; Xiao, H.Y.; Ren, E.H.; Song, Q.S.; Xiang, C.; Lai, X.X.; Lan, J.W.; Jiang, S.X. Bi_2WO_6/Nb_2CT_x MXene hybrid nanosheets with enhanced visible-light-driven photocatalytic activity for organic pollutants degradation. *Appl. Surf. Sci.* **2020,** *505*, 144595.

7.Lu, X.Y.; Che, W.J.; Hu, X.F.; Wang, Y.; Zhang, A.T.; Deng, F.; Luo, S.L.; Dionysiou, D. D. The facile fabrication of novel visible-light-driven Z-scheme $CuInS_2$/Bi_2WO_6 heterojunction with intimate interface contact by in situ hydrothermal growth strategy for extraordinary photocatalytic performance. *Chem. Eng. J.* **2019,** *356*, 819-829.

8.Li, S.J.; Chen, J.L.; Hu, S.W.; Wang, H.L.; Jiang, W.; Chen, X.B. Facile construction of novel Bi_2WO_6/Ta_3N_5 Z-scheme heterojunction nanofibers for efficient degradation of harmful pharmaceutical pollutants.

Chem. Eng. J. **2020,** *402*, 126165.

9.Jia, J.K.; Zhang, X.R.; Jiang, C.Y.; Huang, W.X.; Wang, Y.P. Visible-light-driven nitrogen-doped carbon quantum dots decorated g-C_3N_4/Bi_2WO_6 Z-scheme composite with enhanced photocatalytic activity and mechanism insight. *J. Alloy. Compd.* **2020,** *835*, 155180.

10.Chen, F.; Li, D.; Luo, B.F.; Chen, M.; Shi, W.D. Two-dimensional heterojunction photocatalysts constructed by graphite-like C_3N_4 and Bi_2WO_6 nanosheets: enhanced photocatalytic activities for water purification. *J. Alloy. Compd.* **2017,** *694*, 193-200.

11.Xue, W.J.; Huang, D.L.; Li, J.; Zeng, G.M.; Deng, R.; Yang, Y.; Chen, S.; Li, Z.H.; Gong, X.M.; Li, B. Assembly of AgI nanoparticles and ultrathin g-C_3N_4 nanosheets codecorated Bi_2WO_6 direct dual Z-scheme photocatalyst: an efficient, sustainable and heterogeneous catalyst with enhanced photocatalytic performance. *Chem. Eng. J.* **2019,** *373*, 1144-1157.
